# A Pleasant Method of Administering Castor Oil

**Published:** 1879-06

**Authors:** 


### A PLEASANT METHOD OF ADMINISTERING CASTOR OIL

Consists, says Dr. Starke, in *Berliner Klinische Wochenschrife*, number sixteen, 1879, in mixing the oil with coarse granular sugar until a thick paste is formed. This usually requires about one part of oil to three of sugar. The addition of a small amount of cinnamon powder suffices to give the mass a pleasant taste. The writer finds this candy uniformly successful in children who will rebel against the oil in any other form. The bulk is so great that this mode of administration is almost necessarily confined to children's practice. For adults the addition of compound liquorice powder, in the proportion of one part to two of oil will form a bolus which can be readily swallowed.

